# Supplementary material for: Patient capital and green total factor productivity: Evidence from Chinese listed companies
Source: PLoS One. 2025 Sep 8;20(9):e0331262. doi: 10.1371/journal.pone.0331262 (PMC12416735; doi:10.1371/journal.pone.0331262)
Supplement: Attachment — Submitted filename: 0720Response to Reviewers.docx [file pone.0331262.s002.docx]

**Response Letter**

We sincerely thank the editor and the review team for their constructive comments and valuable suggestions on our manuscript entitled "**Patient capital and green total factor productivity: Evidence from China**". We have carefully considered each comment provided by the reviewers and made detailed revisions accordingly, aiming to enhance both the accuracy and clarity of the paper. Specifically, we have supplemented the theoretical framework regarding the impact mechanisms of patient capital on firms’ green productivity, enriched the empirical analysis with deeper insights into sectoral heterogeneity, and updated the literature review with the most recent references relevant to our topic. We believe these revisions substantially improve the manuscript's rigor and enhance its contribution to the literature, and we hope you will agree.

In addition, we have clearly indicated all major revisions in **bold text** within the revised manuscript to facilitate your review process. Below, we provide detailed, point-by-point responses in a tabular form, specifying how each comment was addressed in our revision. As you will see, we have made every effort to thoroughly respond to your suggestions and hope you find our revision satisfactory.

Thank you once again for your valuable guidance, which has significantly helped us improve the manuscript.

**Responses to the Editor**

| **Comments** | **Responses** |
| --- | --- |
| When submitting your revision, we need you to address these additional requirements.  1. Please ensure that your manuscript meets PLOS ONE's style requirements, including those for file naming. | Thank you for this important reminder. We have carefully checked the manuscript to ensure it fully complies with PLOS ONE's style requirements, including formatting guidelines and file naming conventions. All figures and supporting information files have been named strictly following the instructions provided in the official templates and guidelines from the PLOS ONE website. We have also verified and adjusted the manuscript text, headings, author bylines, affiliations, and reference formatting accordingly. |
| 2. We have amended our Funding Statement to clearly declare all sources of funding or support received during this study, explicitly stating:   This research was supported by the Ministry of Education of China Humanities and Social Sciences Youth Fund Project ‘Refining Local Legislation on Social Credit: An Examination of Fifteen Local Regulations’ (Grant No. 21YJC820023). There was no additional external funding received for this study.  Could you please confirm if this revised funding statement now fully meets the journal’s requirements? | Thank you for your clear instructions regarding the funding statement. We have revised the funding statement as requested to fully disclose all funding sources associated with this study. The updated funding statement is as follows:  This research was supported by the Ministry of Education of China Humanities and Social Sciences Youth Fund Project ‘Refining Local Legislation on Social Credit: An Examination of Fifteen Local Regulations’ (Grant No. 21YJC820023). There was no additional external funding received for this study.  We have included this revised statement within our manuscript and cover letter. |
| 3. We have updated the Competing Interests statement in our manuscript and cover letter to clearly state: “The authors have declared that no competing interests exist.” Could you please confirm if this revised statement now fully aligns with the journal’s requirements, and whether further actions are needed from our side regarding the online submission form? | Thank you for your clear instruction. We confirm that we have revised the Competing Interests statement in our manuscript and included the following statement explicitly in our cover letter:  The authors have declared that no competing interests exist.  We appreciate your assistance in updating the online submission form on our behalf, as you mentioned. If any further actions are needed from our side, please kindly let us know. |
| 4. Regarding the data availability statement, we initially indicated that the data would be made available upon acceptance. Considering your recommendation, we have now established a data sharing plan to ensure that all data will be freely accessible immediately upon acceptance of the manuscript, complying fully with the journal's open data policy. Could you please confirm whether our updated data availability statement adequately addresses the journal's requirements, or if any additional steps are needed? | Thank you for highlighting this important issue. Following your recommendation, we have revised our data availability statement and established a clear data-sharing plan. We confirm that all data related to this manuscript will be freely accessible immediately upon acceptance, fully complying with the journal's open data policy.  If there are additional requirements or further actions needed from our side, please kindly let us know. |
| 5. We have amended the manuscript submission data through the "Edit Submission" portal to include author Luo Huanqi. Could you please confirm whether this addition now correctly meets the journal's submission requirements, or if any further adjustments are necessary? | Thank you for your instruction. We confirm that the manuscript submission data has been amended via the "Edit Submission" system, and author Luo Huanqi has now been included. We have verified this change carefully and ensured that all authorship information aligns correctly with the manuscript.  If there is anything else we should do, please kindly advise. |
| 6. Please amend your authorship list in your manuscript file to include author Luo Huan qi. | Thank you for your careful guidance. We have amended the authorship list in our manuscript file to clearly include author Luo Huanqi. Additionally, we have thoroughly reviewed the reference list to ensure accuracy and completeness. We confirm that no retracted articles are cited in our manuscript, and all references are current and properly formatted.  We have clearly noted these revisions in the rebuttal letter accompanying our revised manuscript. If any additional modifications or clarifications are required, please kindly inform us. |

**References to the Editor**

1.

**Responses to Reviewer #1**

| **Comments** | **Responses** |
| --- | --- |
| When submitting your revision, we need you to address these additional requirements.  1. The existing title is overly broad. To emphasize the study’s firm-level focus, please consider a revision such as “Patient Capital and Green Total Factor Productivity: Evidence from Chinese listed Companies.” You may adopt a different wording, but the final title should explicitly reference firms or enterprises. | Thank you for your insightful suggestion. We agree with your recommendation that the title should clearly reflect the firm-level focus of our study. Accordingly, we have revised the manuscript title to:  Patient Capital and Green Total Factor Productivity: Evidence from Chinese Listed Companies  We believe this new title accurately emphasizes the scope and contribution of our research, clearly highlighting the firm-level analysis as suggested. This revised title has been updated throughout our manuscript and submission materials. |
| 2. throughout the manuscript—including main text, tables, figure captions, and footnotes—replace any full-width double quotation marks with standard half-width English double quotation marks (" ") to ensure consistent formatting. | Thank you for bringing this formatting issue to our attention. We have carefully checked the entire manuscript, including main text, tables, figure captions, and footnotes, and replaced all full-width double quotation marks (“ ”) with standard half-width English quotation marks (" "). This revision ensures consistent formatting throughout the manuscript. |
| 3. Items 36–39 are not in APA format. Convert each to the journal’s required APA style. | Thank you for pointing out the formatting issues in references 36–39. We have now carefully revised these items to fully comply with the journal’s required APA formatting:  Wen, J., Feng, G. F., & Liu, Z. Y. (2011). Heterogeneous debt, company size and R&D input. Journal of Financial Research, 2011(1), 167–181. (In Chinese)  Niu, J. B., Wu, C., & Li, S. N. (2013). Institutional investor types, equity characteristics and voluntary disclosure. Management Review, 2013(3), 48–59. (In Chinese)  Li, Z. G., Zhao, X. B., & Cao, F. (2014). Institutional investor heterogeneity and firm performance: Evidence from Chinese listed companies. Audit & Economy Research, 2014(5), 77–87. (In Chinese)  Wang, X. Y., & Wen, D. Z. (2020). Institutional investor heterogeneity, innovation input and corporate performance: Evidence from GEM‑listed companies. Audit & Economy Research, 2020(2), 98–106. (In Chinese)  These references have been corrected and clearly marked as published in Chinese. |
| 4. The abstract should be limited to 300 words in a single paragraph, with no citations. It must briefly outline the research objectives, data and methods, key results, and policy implications, while avoiding lengthy background information or unnecessary abbreviations. | Thank you for your guidance regarding the abstract. We have revised the abstract carefully according to the journal’s requirements. The updated abstract is now limited to 300 words, formatted as a single paragraph without citations, and concisely highlights our research objectives, data and methods, key results, and policy implications. We have removed unnecessary background information and avoided abbreviations wherever possible. The abstract has been enriched as follows:  This study applies Fisher's investment framework to investigate how patient capital affects firms' green total factor productivity (GTFP). Using panel data from Chinese listed companies over the period 2008–2023, we measure firm-level GTFP by employing a non-radial SBM directional distance function combined with the Malmquist–Luenberger productivity index. Our analysis, based on two-way fixed-effects models and instrumental variable regressions, reveals that an increase in patient capital significantly enhances firms' green total factor productivity. Mechanism analysis indicates that this improvement arises from reduced financing costs as well as increased green R&D investment. Furthermore, the positive impact is particularly pronounced among small and medium-sized enterprises, non-state-owned firms, and pollution-intensive industries. The findings suggest that encouraging the transition from short-term speculative capital toward long-term patient capital can effectively improve firms' environmental efficiency. Thus, policy efforts should be directed towards expanding the supply of patient capital to promote corporate sustainability and accelerate the low-carbon economic transition without compromising economic efficiency.  We believe the revised abstract fully complies with the journal's standards. |
| 5. Replace Roman numerals (I, II, III …) in headings and sub-headings with Arabic numerals (1, 2, 3 …) to align with journal style. | Thank you for bringing this formatting issue to our attention. We have carefully reviewed the manuscript and replaced all Roman numerals (I, II, III, etc.) in headings and sub-headings with Arabic numerals (1, 2, 3, etc.) to align fully with the journal's style requirements. |

**References for Reviewer** **#1**

1.

**Responses to Reviewer #2**

| **Comments** | **Responses** |
| --- | --- |
| When submitting your revision, we need you to address these additional requirements.   1. The current abstract is informative but slightly wordy. Please condense it by tightening phrasing and eliminating non-essential background so key objectives, methods, and findings stand out more clearly. | Thank you for this valuable suggestion. We have carefully revised the abstract by condensing phrasing, removing non-essential background information, and clearly highlighting the key objectives, methods, and findings of our study. We believe the revised abstract is now more concise and effectively emphasizes the core contributions of our research. The abstract has been enriched as follows:  This study applies Fisher's investment framework to investigate how patient capital affects firms' green total factor productivity (GTFP). Using panel data from Chinese listed companies over the period 2008–2023, we measure firm-level GTFP by employing a non-radial SBM directional distance function combined with the Malmquist–Luenberger productivity index. Our analysis, based on two-way fixed-effects models and instrumental variable regressions, reveals that an increase in patient capital significantly enhances firms' green total factor productivity. Mechanism analysis indicates that this improvement arises from reduced financing costs as well as increased green R&D investment. Furthermore, the positive impact is particularly pronounced among small and medium-sized enterprises, non-state-owned firms, and pollution-intensive industries. The findings suggest that encouraging the transition from short-term speculative capital toward long-term patient capital can effectively improve firms' environmental efficiency. Thus, policy efforts should be directed towards expanding the supply of patient capital to promote corporate sustainability and accelerate the low-carbon economic transition without compromising economic efficiency. |
| 2. The current description of the study’s marginal contribution is somewhat verbose. Please focus on the two or three most innovative points and shorten the section accordingly to make the argument more concise and persuasive. | Thank you for your insightful suggestion. We have revised the description of our study’s marginal contributions to focus explicitly on the two to three most innovative aspects. By streamlining this section, we aim to present a more concise, persuasive, and clearly articulated argument regarding the originality and significance of our research. The marginal contributions have been enriched as follows:  This paper offers three key contributions. First, theoretically, we extend Fisher’s two-period investment model to clarify how patient capital, characterized by long-term orientation and higher risk tolerance, enhances firms’ green total factor productivity (GTFP). Second, empirically, we identify two core mechanisms—reduced financing costs and increased green R&D investment—through which patient capital improves firms’ green performance. Third, through heterogeneity analyses, we demonstrate stronger effects for SMEs, non–state-owned enterprises, and pollution-intensive sectors, providing micro-level evidence useful for targeted policy-making in green finance and sustainable firm transition. |
| 3. The section outlining the study’s limitations and future research directions would benefit from greater concision. Please summarize the key points in a more streamlined paragraph. | Thank you for your constructive feedback. We have carefully revised the section discussing the study’s limitations and future research directions. This section now concisely summarizes the key limitations and clearly outlines promising avenues for further investigation. We believe this streamlined paragraph effectively communicates essential points in a clearer and more focused manner. The study's limitations and future research directions have been enriched as follows:  Although this paper systematically explores how patient capital enhances firms’ green total factor productivity (GTFP), it still has several limitations. First, data constraints limit our pollution measures to disclosed waste outputs (industrial wastewater, gas, and solids), which may not fully capture firms’ environmental impacts. Future studies could leverage broader environmental indicators (e.g., greenhouse gas emissions) and emerging data sources (ESG disclosures, satellite data) to refine GTFP measurement. Second, our proxies for patient capital (long-term debt ratios, institutional investor stability) might not fully capture its diverse forms. Future research can incorporate multi-dimensional measures, such as shareholder holding periods, international long-term funding, or policy-driven financing. Third, we mainly examine internal mechanisms without fully considering external governance contexts. Subsequent studies could investigate how environmental policies, market competition, or corporate governance quality shape the effectiveness of patient capital, providing more nuanced guidance for targeted policy-making. |
| 4. Proofread for minor grammar slips (e.g., “reveal that a one–standard-deviation increase … raises,” not “raise”). | Thank you for highlighting this grammar issue. We have thoroughly proofread the manuscript to correct minor grammatical errors, including verb agreement and consistency issues. Specifically, the sentence you mentioned now correctly reads: "Our results reveal that a one-standard-deviation increase in patient capital raises firms' green total factor productivity." We have carefully checked the entire manuscript to ensure accuracy and clarity. |

**References for Reviewer #2**

**Responses to Reviewer #3**

| **Comments** | **Responses** |
| --- | --- |
| When submitting your revision, we need you to address these additional requirements.  1. First, refine the introduction by pinpointing the specific research gap that the current literature has not yet addressed and by stating the core questions this study answers, avoiding overlap with existing reviews. | Thank you for this valuable suggestion. We have refined the introduction by clearly pinpointing the specific research gap that previous literature has overlooked, explicitly highlighting the absence of comprehensive analysis regarding how patient capital affects firms’ green total factor productivity at the micro-level. We have also clarified the core research questions our study addresses, thus ensuring minimal overlap with existing reviews and sharpening our manuscript’s distinct contribution.  Under China’s dual-carbon goals and high-quality development agenda, facilitating enterprises’ green transition has become critical. Green total factor productivity (GTFP), reflecting comprehensive production efficiency under environmental constraints, serves as a key indicator of firms’ sustainable performance. Yet, investments in green innovation often entail substantial upfront costs and long payback periods, posing challenges for short-term-oriented finance. Thus, patient capital, characterized by long-term investment horizons and risk tolerance, emerges as a promising solution to these green financing challenges. These investments entail large capital requirements and long payback periods, making it difficult for "short-sighted" or "quick-profit" capital supply to satisfy the long-term demands of a green transition. However, existing research predominantly explores patient capital’s impacts on conventional corporate outcomes, such as innovation or economic performance, with limited attention to environmental performance at the firm level. Moreover, much literature concentrates on macro-level analyses, neglecting micro-level empirical evidence on how patient capital concretely fosters firms’ green transitions. This gap motivates two core research questions: (1) Can patient capital effectively enhance corporate-level GTFP? (2) If so, through which specific mechanisms does patient capital affect firms’ green performance?  These revisions can be found on page 6 (lines 124–130) and page 7 (lines 135–140) of the revised manuscript. |
| 1. Second, enrich the theory section with the most recent studies on patient capital and green innovation, and end that discussion by clearly stating the incremental contribution of this paper relative to prior work. | Thank you for your valuable advice. We have enriched the theory section by incorporating recent studies on patient capital and green innovation, emphasizing the latest insights and theoretical developments. At the conclusion of this section, we clearly articulate our incremental contribution relative to prior research—specifically highlighting how our study uniquely integrates patient capital into the analysis of firms' green total factor productivity at the micro-level, filling an important research gap in existing literature. The literature review has been enriched as follows:  Recent studies have emphasized patient capital’s role in facilitating ESG performance and green innovation, highlighting its potential for driving sustainable corporate growth [20][21]. At the same time, green innovation research underscores the crucial influence of environmental policies and digital transformation on firms’ productivity [22][23].  However, existing research exhibits two notable shortcomings. First, prior theoretical frameworks rarely integrate patient capital explicitly with firm-level green productivity. Most studies have primarily focused on how patient capital influences conventional economic outcomes, such as corporate R&D and general innovation, with limited consideration of environmental implications. Second, existing empirical research has predominantly emphasized macro-level analyses of environmental regulation and green finance, leaving firm-level evidence underexplored—particularly regarding how patient capital specifically fosters firms’ green transitions and enhances environmental performance. |
| 2. Third, compress the abstract and conclusion so they cover only the four essentials—research purpose, data and methods, key findings, and policy implications—omitting background material that is already well explained in the text. | Thank you for your constructive feedback. We have now compressed both the abstract and conclusion sections, ensuring they concisely cover only the four essential components: the research purpose, data and methods, key findings, and policy implications. We have removed any redundant background information that was already detailed within the main body of the text, thus making these sections clearer and more succinct. The abstract has been enriched as follows:  This study applies Fisher's investment framework to investigate how patient capital affects firms' green total factor productivity (GTFP). Using panel data from Chinese listed companies over the period 2008–2023, we measure firm-level GTFP by employing a non-radial SBM directional distance function combined with the Malmquist–Luenberger productivity index. Our analysis, based on two-way fixed-effects models and instrumental variable regressions, reveals that an increase in patient capital significantly enhances firms' green total factor productivity. Mechanism analysis indicates that this improvement arises from reduced financing costs as well as increased green R&D investment. Furthermore, the positive impact is particularly pronounced among small and medium-sized enterprises, non-state-owned firms, and pollution-intensive industries. The findings suggest that encouraging the transition from short-term speculative capital toward long-term patient capital can effectively improve firms' environmental efficiency. Thus, policy efforts should be directed towards expanding the supply of patient capital to promote corporate sustainability and accelerate the low-carbon economic transition without compromising economic efficiency.  The conclusion section has been enriched as follows:  This study explores how patient capital affects firms' green total factor productivity (GTFP). We theoretically extend Fisher’s two-period investment framework, illustrating patient capital’s advantage in green projects characterized by long investment horizons and uncertain returns. Empirically, using data from China's A-share listed firms (2008–2023) and measuring GTFP via the SBM directional distance function and Malmquist–Luenberger index, we apply fixed-effects regressions to test our hypotheses. Results show that patient capital significantly enhances firms' GTFP by lowering financing costs and increasing green R&D investment. The effect is notably stronger among SMEs, non–state-owned enterprises, and firms in pollution-intensive industries. These findings suggest policymakers should tailor green financing strategies, encouraging patient, long-term investment to effectively support firms’ sustainable transitions and green efficiency improvements. |
| 3. Fourth, add a brief institutional‑background paragraph to the theoretical framework that summarizes the evolution of China’s capital market, the green‑finance policy landscape, and the regulatory impetus of the “dual‑carbon” goals, and explain how these external conditions shape the formation and effects of patient capital. | Thank you for your valuable suggestion. We have added a concise institutional-background paragraph at the beginning of the theoretical framework section. This paragraph briefly summarizes the historical evolution of China’s capital markets, outlines key developments in green-finance policies, and highlights the regulatory drivers related to China’s "dual-carbon" goals (carbon peaking and carbon neutrality). Additionally, we explicitly clarify how these external institutional and policy conditions have shaped the formation, availability, and effectiveness of patient capital in influencing firms' green total factor productivity. The theoretical section has been enriched as follows:  Policy background  Over the past three decades, China's capital market has gradually transitioned from a predominantly short-term financing structure toward a more diversified and long-term-oriented investment and financing system. Recently, with the progressive advancement of green-finance policies and the clear regulatory impetus provided by the national "dual-carbon" goals (carbon peaking and carbon neutrality), the external financing environment and policy directions faced by enterprises have changed significantly. Regulators have actively encouraged the cultivation and expansion of long-term, patient capital, prompting investors to increasingly emphasize environmental, social, and governance (ESG) performance and support corporate green innovation through sustained investment horizons and risk-sharing mechanisms. Thus, the formation, allocation, and effectiveness of patient capital have been shaped profoundly by these external institutional conditions, laying a solid foundation for our analysis of the relationship between patient capital and firms' green total factor productivity. |
| 4. Fifth, strengthen the explanation of the positive feedback loop between lower financing costs and green R&D: clarify how patient capital reduces refinancing risk, encourages longer‑horizon green projects, and in turn attracts additional patient capital, creating a virtuous cycle. | Thank you for your insightful suggestion. We have strengthened our theoretical mechanism section by clearly illustrating the positive feedback loop between reduced financing costs and increased green R&D. Specifically, we now explicitly describe how patient capital lowers firms’ refinancing risks, enabling enterprises to confidently undertake longer-horizon green projects. Successful implementation of these green initiatives then enhances firm credibility and stability, attracting additional patient capital and thus forming a self-reinforcing virtuous cycle. This expanded explanation clarifies how patient capital creates enduring conditions conducive to sustainable green innovation. |
| 5. Sixth, make the policy section more actionable by proposing concrete tools—such as tax incentives, disclosure guidelines, and long‑term investment rewards—and tailoring recommendations to firms of different sizes and ownership types. | Thank you for your constructive feedback. We have enhanced the policy section by recommending specific and actionable tools, including tax incentives to encourage patient capital investment, clear disclosure guidelines to improve transparency around firms’ environmental performance, and reward mechanisms for long-term investors. Additionally, we have tailored policy suggestions to the varying needs of firms by explicitly differentiating recommendations according to firm size (small, medium, large) and ownership structure (state-owned vs. non–state-owned enterprises). These targeted and actionable measures should help policymakers and financial institutions effectively support diverse firms in achieving sustainable green transitions. The policy recommendations have been enriched as follows:  Building on the crucial role patient capital plays in enhancing firms' green total factor productivity (GTFP), this paper proposes three actionable policy recommendations aligned with China's "dual-carbon" strategy and high-quality development goals:  First, proactively expand the supply of patient capital by encouraging long-term institutional investors to enter the green investment sector. Policymakers could introduce targeted tax incentives, direct subsidies, and preferential policies for pension funds, insurance companies, and philanthropic foundations to stimulate their participation in long-term green projects.  Second, financial institutions should innovate and offer long-term green financial instruments better aligned with the duration and risks of environmental projects. Banks and financial markets could develop products such as Green Development Bonds and Asset-Backed Securities (ABS) with maturities exceeding ten years. Additionally, implementing guarantee schemes or insurance-backed risk-sharing mechanisms can help enterprises mitigate technological and policy risks associated with green transitions.  Third, tailor green finance mechanisms specifically to address the diverse needs of firms based on their size, ownership, and industry characteristics. For small and medium-sized enterprises (SMEs), measures such as green loan guarantee insurance, SME-specific green bonds, and reduced collateral requirements can effectively alleviate their financing constraints. Non–state-owned enterprises (non-SOEs) would benefit from fiscal incentives, flexible collateral valuation approaches, and simplified administrative procedures. Firms in pollution-intensive industries could receive targeted support through dedicated green transformation funds, sector-specific subsidies, and public-private collaborative platforms.  Overall, leveraging patient capital's stability and risk tolerance through these differentiated and targeted tools will effectively channel financial resources toward sustainable environmental innovations, accelerating China’s ecological progress and contributing significantly to the nation's dual-carbon targets. |
| 6. Seventh, integrate a corporate‑governance perspective by discussing how disclosure practices, performance metrics, and board incentives can work in tandem with patient capital to advance effective green transitions. | Thank you for your insightful suggestion. We have integrated a corporate-governance perspective into our discussion, explicitly highlighting how enhanced disclosure practices, clearly defined green-performance metrics, and targeted board-level incentives can complement patient capital. We emphasize that transparent environmental disclosures improve investor confidence, robust performance metrics ensure accountability in green transitions, and aligning board incentives with long-term sustainability goals reinforces patient capital’s effectiveness. This integrated approach provides a comprehensive governance framework, further enhancing the practical implications of our study for effective corporate green transitions. |
| 7. Eighth, standardize terminology and formatting throughout the manuscript, eliminate any Chinese–English mixtures, and ensure that all references fully comply with the journal’s style guidelines. | Thank you for highlighting this essential formatting issue. We have now conducted a thorough review of the manuscript to standardize all terminology and formatting. Specifically, we eliminated any inconsistent usage or mixing of Chinese and English terms, ensuring the entire text is consistently presented in clear English. Additionally, we carefully reviewed and adjusted the entire reference list to ensure full compliance with the journal’s style guidelines. |

**References for Reviewer #3**

1. Trudeau, D. (2018). Integrating social equity in sustainable development practice: Institutional commitments and patient capital. Sustainable cities and society, 41, 601-610.
2. Eliwa, Y., & Elmaghrabi, M. E. (2025). Investment horizons and ESG decoupling: Distinct roles of long-term and short-term institutional investors. Economics Letters, 247, 112207.
3. Yan, Z., Yu, Y., Du, K., & Zhang, N. (2024). How does environmental regulation promote green technology innovation? Evidence from China's total emission control policy. Ecological Economics, 219, 108137.
4. Yan, X., & He, T. (2024). Wish fulfilment or wishful thinking?–Assessing the outcomes of China's pilot carbon emissions trading scheme on green economy efficiency in China's cities. Energy Policy, 192, 114261.
